# Clinical activity of 9-ING-41, a small molecule selective glycogen synthase kinase-3 beta (GSK-3β) inhibitor, in refractory adult T-Cell leukemia/lymphoma

**DOI:** 10.1080/15384047.2022.2088984

**Published:** 2022-07-09

**Authors:** Andrew Hsu, Kelsey E. Huntington, Andre De Souza, Lanlan Zhou, Adam J. Olszewski, Nirav P. Makwana, Diana O. Treaba, Ludimila Cavalcante, Francis J. Giles, Howard Safran, Wafik S. El-Deiry, Benedito A. Carneiro

**Affiliations:** a Division of Hematology/Oncology, Brown University and the Lifespan Cancer Institute, Providence, RI, USA; b Legorreta Cancer Center at Brown University, The Warren Alpert Medical School, Brown University, Providence, RI, USA; c Department of Pathology and Laboratory Medicine, The Warren Alpert Medical School, Brown University, Providence, RI, USA; d Department of Radiology, The Warren Alpert Medical School, Brown University, Providence, RI, USA; e Actuate Therapeutics, Fort Worth, Tx, USA

**Keywords:** ATLL, GSK-3, GSK-3b, 9-ING-41, glycogen synthase kinase 3

## Abstract

GSK-3β is a serine/threonine kinase implicated in tumorigenesis and chemotherapy resistance. GSK-3β blockade downregulates the NF-κB pathway, modulates immune cell PD-1 and tumor cell PD-L1 expression, and increases CD8 + T cell and NK cell function. We report a case of adult T-cell leukemia/lymphoma (ATLL) treated with 9-ING-41, a selective GSK-3β inhibitor in clinical development, who achieved a durable response. A 43-year-old male developed diffuse lymphadenopathy, and biopsy of axillary lymph node showed acute-type ATLL. Peripheral blood flow cytometry revealed a circulating clonal T cell population, and CSF was positive for ATLL involvement. After disease progression on the 3^rd^ line of treatment, he started treatment with 9-ING-41 monotherapy in a clinical trial (NCT03678883). CT imaging after seven months showed a partial response. Sustained reduction of peripheral blood ATLL cells lasted 15 months. Treatment of patient-derived CD8 + T cells with 9-ING-41 increased the secretion of IFN-γ, granzyme B, and tumor necrosis factor-related apoptosis-inducing ligand (TRAIL). In conclusion, treatment of a patient with refractory ATLL with the GSK-3β inhibitor 9-ING-41 resulted in a prolonged response. Ongoing experiments are investigating the hypothesis that 9-ING-41-induced T cell activation and immunomodulation contributes to its clinical activity. Further clinical investigation of 9-ING-41 for treatment of ATLL is warranted.

## Introduction

Adult T-cell leukemia/lymphoma (ATLL) is a rare and aggressive mature T cell neoplasm associated with human T-cell lymphotropic virus (HTLV-1) infection^1^. Despite significant advances in our understanding of disease pathogenesis, the diagnosis carries a dismal prognosis and there are limited effective therapies. Commonly used therapeutic agents include antiretrovirals in combination with interferon-alpha, chemotherapy, stem cell transplant, and lenalidomide.^2^ The treatment responses to these strategies frequently lack durability and novel treatments are urgently needed. Here, we present a case of ATLL in a male patient that experienced a significant and durable response to 9-ING-41 monotherapy, a potent small molecule inhibitor of glycogen synthase kinase-3 beta (GSK-3β), following three lines of prior treatments.

## Case presentation

A 43-year-old male with history of hypertension presented to the emergency department with a 1-week history of headache, abdominal pain, nausea, and vomiting associated with hypercalcemia (18 mg/dL; normal[n]: 8.5–10.5 mg/dL). Laboratory evaluation revealed leukocytosis (13.7 x 10^9^/L; n: 3.5–11.0 x 10^9^/L) with an absolute lymphocytosis (7.1 x 10^9^/L; n: 1.0–4.0 x 10^9^/L), anemia (hemoglobin 11.3 g/dL; n: 13.5–16 g/dL), thrombocytopenia (124 x 10^9^/L; normal: 150–400 x 10^9^/L), elevated lactate dehydrogenase (LDH; 316 IU/L; n: 110–220 IU/L), and renal and liver functions within normal limits.

Work-up for hypercalcemia revealed suppression of parathyroid hormone (PTH; 6 pg/dL; n: 18–80 pg/dL), normal PTH-related protein (PTH-RP; 20 pg/dL; n: 0–23 pg/dL); decreased 25-hydroxyvitamin D (9.2 ng/d; n: 30–100 ng/dL), 1,25-dihydroxyvitamin D total, D2, and D3 (< 8 pg/mL; n: 19.9–79.3 pg/dL). These results were suggestive of hypercalcemia secondary to malignancy. Screening for human immunodeficiency virus (HIV-1/-2), hepatitis B and C was negative. Computerized tomography (CT) of the chest revealed bilateral axillary and upper abdominal (gastrohepatic ligament, celiac, peripancreatic) lymphadenopathy (largest lymph node measuring 12 mm). Magnetic resonance imaging (MRI) of the brain and spine showed diffuse signal abnormality involving the marrow without evidence of leptomeningeal disease.

**Figure 1.**
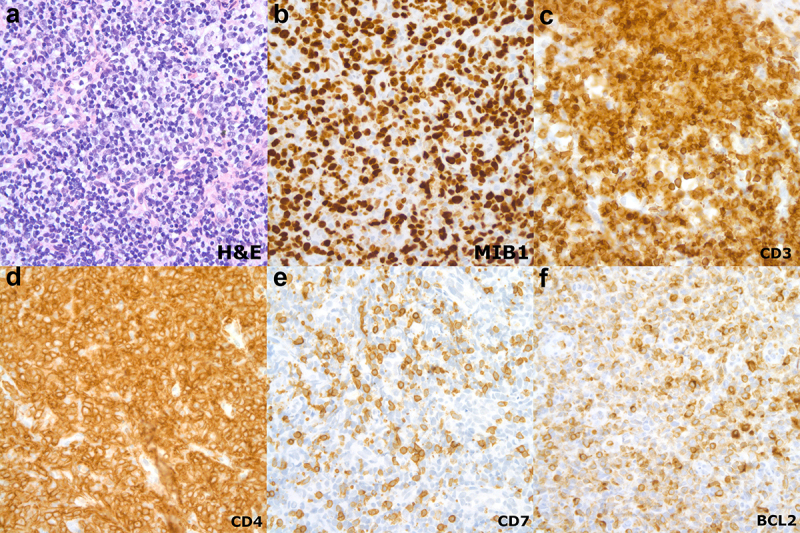
Right axillary lymph node biopsy at diagnosis. (a) Diffuse polymorphic lymphoid population comprised of small to medium sized lymphocytes with irregular nuclei and indistinct nucleoli with a minor subset of large lymphoid cells with irregular nuclei and conspicuous nucleoli (hematoxylin and eosin stain x50 objective, immersion oil). (b) proliferation rate is approximately 70–80% (MIB1 antibody, x50 objective, immersion oil); (c-f) A predominant T-lymphoid population was detected by immunohistochemistry with expression of CD3, CD4 and with significant loss of CD7 and BCL2 (x50 objective, immersion oil) .

A right axillary lymph node core biopsy showed a diffuse polymorphic lymphoid population comprised of small to medium sized lymphocytes with irregular nuclei and indistinct nucleoli with a minor subset of large lymphoid cells with irregular nuclei and conspicuous nucleoli. A predominant T-lymphoid population was detected by immunohistochemistry (IHC) with expression of CD3, CD4, CD43 and CD5, variable expression of MUM1, and loss of CD7 and Bcl2 (Figure 1). Approximately 30% of the lymphoid population expressed c-myc with proliferation rate of 70–80% (MIB1 antibody). Epstein-Barr virus-encoded RNA (EBER) in-situ hybridization stain was negative. Polymerase chain reaction (PCR) for human T-cell leukemia virus-1 (HTLV-1) was positive, but negative for HTLV-2. Molecular studies were positive for T-cell receptor beta and gamma gene rearrangements. Flow cytometry immunophenotypic analysis of peripheral blood showed that 85% of the circulating lymphoid cells were neoplastic T-lymphoid cells co-expressing CD4, CD5, CD7, with dim surface CD3 positivity, and negative for CD7 and CD8 (5.2 x 10^9^/L). Flow cytometry from the cerebrospinal fluid (CSF) also detected a neoplastic T-lymphoid population (32% of the lymphoid cells) CD3 and CD4 positive, and CD7 negative. These findings were consistent with the diagnosis of adult T-cell leukemia/lymphoma (ATLL) with CSF involvement (acute type per Shimoyama criteria).^3^

## Treatment course

Prior to the final pathology result the patient started a four-day course of dexamethasone (40 mg/day) given suspicion for underlying lymphoma contributing to symptomatic hypercalcemia. Once the diagnosis of ATLL was confirmed, the patient started treatment with zidovudine (AZT; 300 mg every 8 hours), lamivudine (150 mg BID), interferon-alpha (INF-α; 5 million units s.c. daily) associated with weekly intrathecal methotrexate, cytarabine and hydrocortisone given the CSF involvement. He received this therapy throughout his initial 4-week hospitalization and at time of discharge, INF-α was transitioned to pegylated INF-α (1.5 mcg/kg weekly). At 9 weeks of therapy, analysis of the CSF showed low cellularity and clonal T-lymphoid population was undetectable by PCR. At 10 weeks of therapy, the patient achieved a partial response by Japan Clinical Oncology Group (JCOG) response criteria [1] – peripheral flow cytometry at 6 weeks had shown involvement of his ATLL with a reduction in the absolute count to 0.19 x10^9^/L and LDH (204 IU/L). At 12 weeks of therapy, given persistently negative CSF, intrathecal therapy was decreased to every four weeks and the patient was referred for evaluation for allogeneic stem cell transplant, but he was considered a poor candidate given his frailty and deconditioning. CT imaging showed greater than 50% reduction in size of his lymphadenopathy. AZT, lamivudine and raltagravir were continued for maintenance.

At 8 months of therapy, a positron emission tomography (PET) scan showed new cervical, axillary, abdominal, inguinal and iliac lymphadenopathy suggestive of progressive disease which was confirmed with a biopsy of a left inguinal lymph node. Next-generation sequencing (NGS) showed a *DNMT3A* mutation (2.8% variant allele frequency [VAF]). Peripheral blood flow cytometry confirmed persistent involvement of his ATLL (0.15 x 10^9^/L). The patient received second-line therapy with mogamulizumab (monoclonal antibody against CC chemokine receptor 4) with raltagravir. After 5 weekly infusions, the patient developed a diffuse, desquamating, and erythrodermic skin rash consistent with mogamulizumab-related exanthem leading to treatment discontinuation. CT scans one month later showed progression of diffuse adenopathy. The patient started third-line treatment with lenalidomide (25 mg daily) and raltagravir; however, after a month of therapy, the patient developed a desquamating rash which improved after holding lenalidomide. The rash persisted despite dose reduction of lenalidomide leading to discontinuation of lenalidomide.

**Figure 2.**
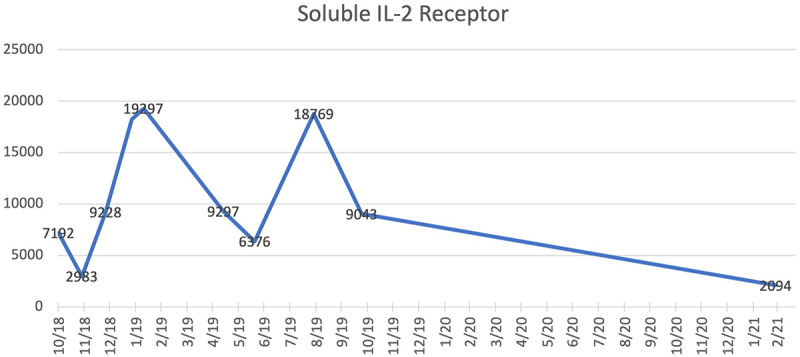
sIL-2 r Serum Concentration: sIL-2 r serum concentration (7,192 ng/ml) at first progression on 10/2018 leading to transition to second-line mogamulizumab; second progression (19,297 ng/ml) on 01/2019 leading to transition to third-line lenalidomide; sIL-2 r serum concentration (9,043 ng/ml) one month prior to initiation of 9-ING-41; and sIL-2 r serum concentration (2,094 ng/ml) at best response 15 months after initiation of 9-ING-41.

Twenty-two months after diagnosis, as a fourth-line therapy in the presence of actively progressing disease, the patient was enrolled a phase I/II study investigating the safety and efficacy of 9-ING-41, a selective small molecule inhibitor of glycogen synthase kinase-3 beta (GSK-3β) as monotherapy or in combination with chemotherapy in refractory solid tumors and hematological malignancies (NCT03678883). The patient started treatment with single-agent 9-ING-41 (12.37 mg/kg IV twice weekly: 21-day cycle). Peripheral flow cytometry prior to enrollment showed persistent involvement of ATLL (0.25x10^9^/L). He tolerated the treatment well without significant adverse effects. CT imaging after 2 cycles showed stable disease which was confirmed on CT imaging after 5 and 7 cycles of therapy. CT imaging after 10 cycles showed partial response by RECIST Criteria and continued response after 19 cycles of treatment. Response to the treatment was also demonstrated by marked decrease in the serum concentration of soluble IL-2 receptor (sIL-2 r), which has been associated clinical activity of ATLL and can serve as a surrogate for response to therapy.^4^ sIL-2 r serum concentration one month prior to treatment with 9-ING-41 was 9,043 ng/ml and decreased to 2,094 ng/ml after 19 cycles of treatment (Figure 2).

During cycle 20, the patient experienced abdominal pain, nausea, vomiting and was diagnosed with recurrence of hypercalcemia (15.6 mg/dL) and CT scans revealed bilateral axillary lymphadenopathy with new hepatic lesions consistent with disease progression. Treatment was switched to arsenic trioxide followed by one cycle of etoposide, doxorubicin, vincristine, cyclophosphamide, prednisone (EPOCH) without clinical benefit. The patient transitioned to hospice and passed away approximately 6 weeks from time of disease progression. Next-generation sequencing (NGS) of peripheral blood specimen showed a new *TP53* mutation (c.637C>T; 70.3% VAF) and copy number analysis showed deletion of *ATM* (on chromosome 11q) and *TP53* (chromosome 17q) and gain of *NOTCH1, AKL1* (chromosome 9q).

**Figure 3.**
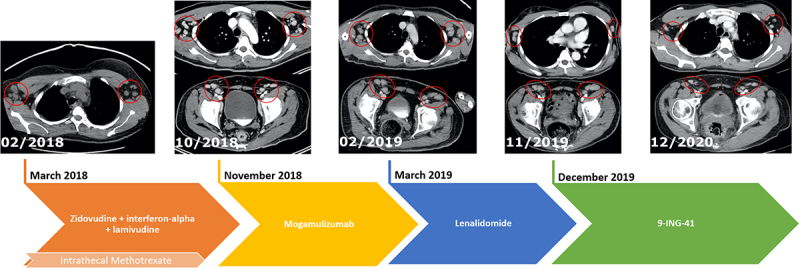
Timeline of Therapy with Correlating CT Imaging. (02/2018): Imaging at diagnosis showing enlarged bilateral axillary lymph nodes; (10/2018): progression of disease with increase in size of axillary and inguinal lymph nodes leading to transition to second-line mogamulizumab; (02/2019): Increase in size of axillary and inguinal lymph nodes leading to transition to third-line lenalidomide; (11/2019): persistently enlarged axillary and inguinal lymph nodes prior to start treatment with 9-ING-41, a selective small molecule inhibitor of GSK-3β; (12/2020): response with improvement of axillary and inguinal lymph nodes after 1 year of therapy with 9-ING-41.

In summary, our patient with refractory ATLL experienced a significant and durable (15 months) response to 9-ING-41 following 3 prior lines of treatments (Figure 3). Disease progression was associated with a newly acquired *TP53* mutation.

## Discussion

ATLL is an aggressive malignancy of mature, activated T-cells that emerges in 2–5% of subjects with HTLV-1 infection.^1^ ATLL is endemic in Africa, regions of South America, Japan, the Caribbean, and northern Iran. Its clinical course, prognosis, and management are dependent upon the four clinical variants: acute, lymphoma-type, chronic, and smoldering.^2^ Particularly aggressive variants include “acute ATLL” which describes a leukemic presentation (60% of the cases), and “lymphoma-type ATLL” which is associated with lymph node enlargement without peripheral blood involvement (20% of the cases) .^5^

ATLL is characterized by the clonal integration of the HTLV-1 provirus in mature, activated T-cells. Expansion of these T-cells results from the expression of the viral oncoprotein Tax-1, which in turn activates transcription factors (e.g. cAMP-dependent transcription factor, nuclear factor kappa-B [NF-κB]), inhibits apoptosis (e.g. repression of p53), disrupts cell cycle control, and interferes with genetic stability (DNA polymerase β, proliferating cell nuclear antigen, and the mitotic spindle-assembly checkpoint protein MAD1) .^6–10^ It is still unclear why only 2–5% of patients infected with HTLV-1 develop ATLL. Virus genetic variants, host HLA genotype, immune escape mediated by overexpression of PD-L1 in ATLL may contribute to ATLL’s development and progression.^11,12^ In a cohort of 22 patients with ATLL, 22% of patients had increased PD-L1 expression in ATLL cells. PD-1 expression was also increased in HTLV-1-specific cytotoxic T-lymphocytes which are associated with T cell exhaustion. In fact, anti-HTLV-1 Tax CD8 + T cells isolated from patients with ATLL had diminished immune function. Treatment of HTLV-1 specific CD8+ cells with anti-PD-L1 antibody increased the production of IFN-γ, TNF-α, and expression of CD107 suggestive of reactivation of exhausted T cells.^13^ These results suggest that increased expression of PD-1/PD-L1 checkpoint might contribute to chronic HTLV-1 infection, immune evasion and ultimately development of ATLL representing a potential therapeutic target to enhance immune response against viral infections.^14^

The very poor prognosis of patients with aggressive variants is attributed to intrinsic resistance to chemotherapy and immunosuppression from HTLV-1. In the largest retrospective study of 1,594 Japanese patients with aggressive variants of ATLL, median overall survival (mOS) was 8–11 months with a 4-year survival rate of 11–16% with first-line chemotherapy.^2^ The outcomes of patients with aggressive variants improved with allogeneic stem cell transplant (mOS 14 months from time of diagnosis; 4-year OS 26%). Alternative first-line treatments of acute ATLL include the combination of INF-α with AZT, an antiretroviral agent, based upon phase II studies.^15^ In relapsed/refractory disease, mogamulizumab and lenalidomide have limited clinical activity with median progression free survival (mPFS) of 4–5 months.^16,17^

Glycogen synthase kinase-3 beta (GSK-3β), a serine/threonine protein kinase, regulates multiple intracellular signaling pathways involved in carcinogenesis and modulates T cell PD-1 expression as well as tumor cell PD-L1 expression.^18–20^ The inactivation of GSK-3 leads to a T-bet-mediated downregulation of PD-1 expression on T cells, resulting in increased CD8 + T cell cytotoxicity.^21^ Moreover, GSK-3β has been shown to induce phosphorylation-dependent proteasomal degradation of PD-L1 by β-TrCP.^22^ GSK-3β has been implicated in cancer cell proliferation and survival in various malignancies – its aberrant expression is thought to serve as either a tumor suppressor by priming oncogene products to proteasome destruction or as a pro-oncogene, mainly through proliferative pathways such as Wnt.^23,24^ GSK-3β positively regulates human cancer cell survival through regulation of NF-κB-mediated expression of anti-apoptotic molecules.^25^ For instance, in multiple myeloma, GSK-3 plays a pro-survival role in myeloma cells through the constitutive activation of the noncanonical NF-κB pathway. GSK-3 interacts and degrades p100, an inhibitor of the noncanonical NF-κB signaling. Inhibition of GSK-3 prevents p100 degradation and allows for p100-mediated suppression of the noncanonical NF-κB pathway and myeloma cell death.^26^ These findings coupled with anti-tumor activity in models of several malignancies supported the clinical development of 9-ING-41 for treatment of solid organs and hematological malignancies. The agent has demonstrated a favorable safety profile and durable responses in adult patients with refractory malignancies including melanoma and pancreatic cancer.^27^

The clinical activity of 9-ING-41 in ATLL observed in this case may in part derive from its immune modulation properties including downregulation of checkpoints PD-1 and lymphocyte activation gene-3 (LAG-3) .^21,28,29^ GSK-3 blockage enhances T-bet (*Tbx21)* transcription that in turn suppresses *Pdcd1* gene transcription which ultimately decreases PD-1 expression on CD8 T-cells.^21^ GSK-3 blockade also upregulates interferon gamma (IFN-γ) and granzyme B enhancing an anti-tumor immune response via CD8 T-cells.^20^ 9-ING-41 increases NK and T-cell effector function in models of colon cancer.^30^ GSK-3 inhibition increases proliferation of T-cells and reduces the growth of melanoma tumors in mice.^28^ These results together with the clinically meaningful and durable response in our patient led to our hypothesis that 9-ING-41 induced activation of CD8 + T cells resulting in cell killing of ATLL malignant cells.

**Figure 4.**
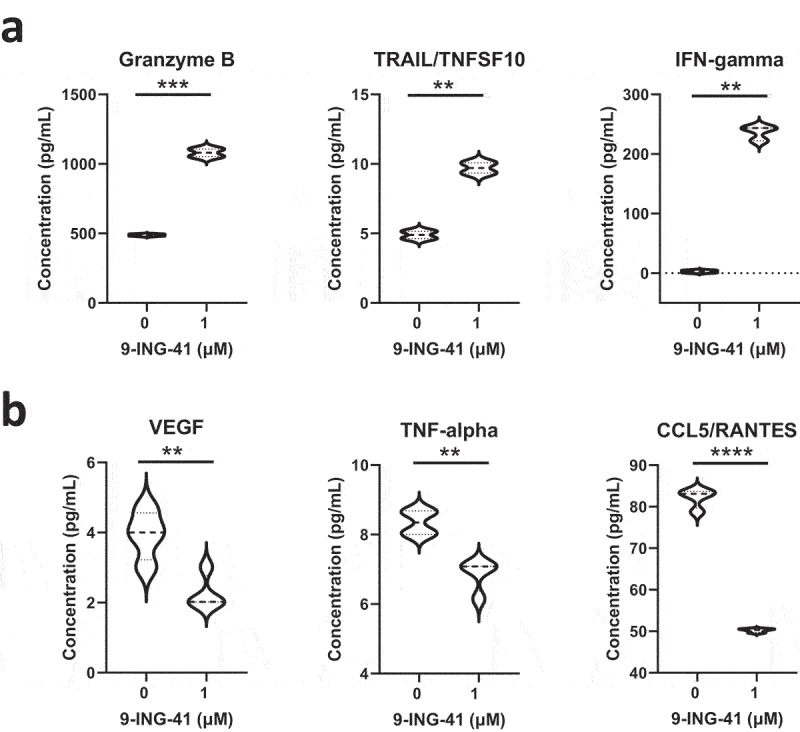
Treatment of patient-derived CD8 + T cells with 9-ING-41 increases granzyme B, TRAIL, and IFN-gamma secretion while decreasing VEGF, TNF-alpha, and CCL5/RANTES concentrations in cell culture supernatant in comparison to control groups. Patient-derived CD8+ cytotoxic T cells were treated with 9-ING-41 for 48 hours (1 µM) or control (DMSO) and cytokine concentration was measured in cell culture supernatants. (a) Violin plots representing cytokine concentrations that increased post-treatment. (b) Violin plots representing cytokine concentrations that decreased post-treatment. Statistical significance was calculated with GraphPad Prism 9.3.1 using unpaired t tests and is denoted on each plot as follows: P > .05 = n.s., P ≤ .05 = *, P ≤ .01 = **, P ≤ .001 = ***, and P ≤ .0001 = **** (N = 3).

We collected peripheral blood during the patient’s best response at 15 months, corresponding with his lowest concentration of sIL-2 r in the peripheral blood, and isolated CD8+, non-ATLL T cells for further analysis. We then treated these CD8+ cells *ex vivo* with 9-ING-41 and assessed changes in cytokine, chemokine, and growth factor profiles in the cell culture supernatant using Luminex (LX200) technology and a custom cytokine panel.^31^ In agreement with the aforementioned observation and supporting the activation of CD8 + T cells, treatment of patient-derived CD8 + T cells with 9-ING-41 increased the concentration of IFN-γ, granzyme B, and tumor necrosis factor-related apoptosis-inducing ligand (TRAIL) in comparison to control groups treated with DMSO (Figure 4). IFN-γ is a key effector cytokine in immunity and upregulates major histocompatibility complex (MHC) molecules and the machinery involved in antigen processing and presentation.^32^ This upregulation of cell surface MHC class I by IFN- γ is essential for cytotoxic T cell activation, and thus, the host response to tumor cells.^33^ Granzyme B has long been known as a pro-apoptotic protease that is expressed by both cytotoxic lymphocytes and natural killer cells. Activation of CD8 + T cells induces the expression of granzyme B which is delivered, along with perforin, to target cells and induces apoptosis.^34^ 9-ING-41 also increased the production of TRAIL by CD8+ T cells. Expression of TRAIL by immune cells induces apoptosis in tumor cells.^34^ In fact, GSK-3 inhibition has been shown to enhance both tumor necrosis factor-alpha- and TRAIL-induced apoptosis in pancreatic cell lines.^35^ These results support the hypothesis that 9-ING-41 treatment promoted activation of patient’s CD8 + T cells that might have contributed to the killing or suppression of ATLL cells. The anti-tumor activity of 9-ING-41 in this case of ATLL might be also associated with the modulation of the NK-kB pathway, which is involved in the pathogenesis of lymphoproliferative disorders,^36,37^ or activation of NK cells induced by 9-ING-41 as recently reported in co-culture experiments.^30^ The inability to isolate TALL cells from the patient limited the investigation of 9-ING-41 direct cytotoxicity in these cells *in vitro*. However, our results suggest that anti-tumor activity of 9-ING-41 in this case of ATLL might have derived from indirect modulation of immune cells rather than direct cytotoxicity. Importantly, this hypothesis is being tested in ongoing experiments investigating possible mechanisms, including how small-molecule inhibition of GSK-3 can induce anti-tumor immunity, in parallel with efforts to enroll patients with ATLL in the ongoing clinical trial with 9-ING-41.

9-ING-41 downregulates NF-κB and its target genes *cyclin D1, Bcl-2*, anti-apoptotic protein (*XIAP*) and B-cell lymphoma-extra large (*Bcl-XL*) leading to inhibition of tumorigenesis.^24^ 9-ING-41 enhanced apoptosis of aggressive B-cell lymphoma lines through downregulation of anti-apoptotic mechanisms. This anti-tumor activity was documented with 9-ING-41 monotherapy with enhancement of effect when combined with venetoclax, a Bcl-2 inhibitor, and BAY-1143572, a CDK-9 inhibitor.^38^ In ATLL, the continual activation and dysregulation of NF-κB through Tax-1 that contributes to tumorigenesis, provides another potential mechanism to explain that clinical activity of 9-ING-41 which warrants further mechanistic studies involving a larger patient cohort.^39^

In view of this patient’s exceptional response and preclinical results suggesting that 9-ING-41 promotes immune activation of CD8 + T cells, an expansion cohort of patients with refractory ATLL was added to the ongoing Phase 1/2 study of 9-ING-41. Ongoing *in vitro* and *in vivo* experiments, including immunocompetent syngeneic murine models of cancer, are investigating the impact of 9-ING-41 in the anti-tumor immune response.

## Data Availability

The authors confirm that the data supporting the findings of this study are available within the article and/or its supplementary materials.
